# Recent advances in transition-metal-catalyzed intermolecular carbomagnesiation and carbozincation

**DOI:** 10.3762/bjoc.9.34

**Published:** 2013-02-11

**Authors:** Kei Murakami, Hideki Yorimitsu

**Affiliations:** 1 Department of Chemistry, Graduate School of Science, Kyoto University, Sakyo-ku, Kyoto 606-8502, Japan; 2 Japan Science and Technology Agency, Department of Research Projects (ACT-C), Tokyo 102-0076, Japan

**Keywords:** alkene, alkyne, carbomagnesiation, carbometalation, carbozincation, transition metal

## Abstract

Carbomagnesiation and carbozincation reactions are efficient and direct routes to prepare complex and stereodefined organomagnesium and organozinc reagents. However, carbon–carbon unsaturated bonds are generally unreactive toward organomagnesium and organozinc reagents. Thus, transition metals were employed to accomplish the carbometalation involving wide varieties of substrates and reagents. Recent advances of transition-metal-catalyzed carbomagnesiation and carbozincation reactions are reviewed in this article. The contents are separated into five sections: carbomagnesiation and carbozincation of (1) alkynes bearing an electron-withdrawing group; (2) alkynes bearing a directing group; (3) strained cyclopropenes; (4) unactivated alkynes or alkenes; and (5) substrates that have two carbon–carbon unsaturated bonds (allenes, dienes, enynes, or diynes).

## Introduction

Whereas direct transformations of unreactive carbon–hydrogen or carbon–carbon bonds have been attracting increasing attention from organic chemists, classical organometallic reagents still play indispensable roles in modern organic chemistry. Among the organometallic reagents, organomagnesium and organozinc reagents have been widely employed for organic synthesis due to their versatile reactivity and availability. The most popular method for preparing organomagnesium and organozinc reagents still has to be the classical Grignard method [1], starting from magnesium or zinc metal and organic halides [2–7]. Although the direct insertion route is efficient and versatile, stereocontrolled synthesis of organomagnesium or organozinc reagents, especially of alkenyl or alkyl derivatives, is always difficult since the metal insertion process inevitably passes through radical intermediates to lose stereochemical information [5,8]. Halogen–metal exchange is a solution for the stereoselective synthesis [9–13]. However, preparation of the corresponding precursors can be laborious when highly functionalized organometallic species are needed. Thus, many organic chemists have focused on carbometalation reactions that directly transform simple alkynes and alkenes to structurally complex organometallics with high stereoselectivity.

In general, carbon–carbon multiple bonds are unreactive with organomagnesium and organozinc reagents. Hence, limited substrates and reagents could be employed for uncatalyzed intermolecular carbometalation. Naturally, many groups envisioned transition-metal-catalyzed carbometalation reactions that directly convert alkynes and alkenes to new organomagnesium and organozinc reagents [14–33]. The resulting organomagnesium and organozinc intermediates have versatile reactivity toward various electrophiles to provide multisubstituted alkenes and alkanes. Thus, carbomagnesiation and carbozincation reactions are highly important in organic synthesis. Although intramolecular carbomagnesiation and -zincation [15] and intermolecular carbocupration with stoichiometric copper reagents has been well established [14,18,25], catalytic intermolecular carbomagnesiation and carbozincation are still in their infancy.

**Scheme 1:**
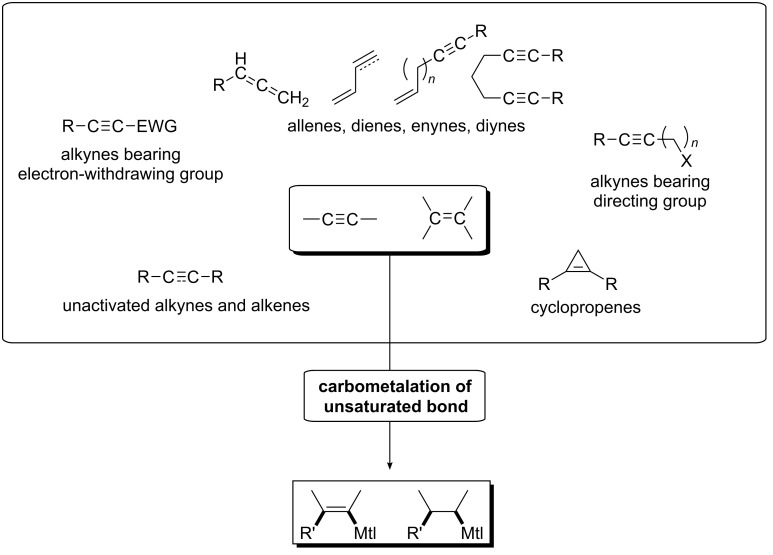
Variation of substrates for carbomagnesiation and carbozincation in this article.

This article includes the advances in transition-metal-catalyzed intermolecular carbomagnesiation and carbozincation reactions that have been made in the past 15 years, promoting the development of these potentially useful technologies. The contents are categorized by the substrates (Scheme 1): (1) alkynes bearing an electron-withdrawing group; (2) alkynes bearing a directing group; (3) cyclopropenes; (4) unactivated alkynes or alkenes; and (5) substrates that have two carbon–carbon unsaturated bonds (allenes, dienes, enynes, or diynes).

## Review

### Carbomagnesiation and carbozincation of electron-deficient alkynes

**Scheme 2:**
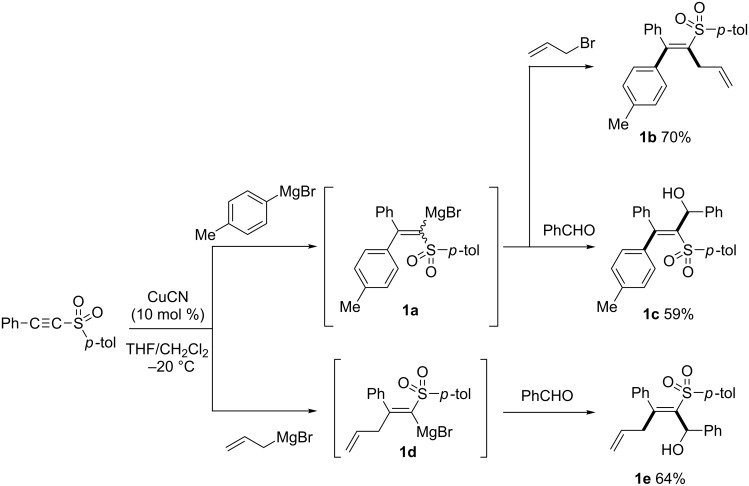
Copper-catalyzed arylmagnesiation and allylmagnesiation of alkynyl sulfone.

Since conjugate addition reactions of organocuprates with alkynyl ketones or esters have been well established [14,34–37], alkynes bearing an electron-withdrawing group other than carbonyl have been investigated recently [25,38]. The Xie, Marek, and Tanaka groups have been interested in copper-catalyzed carbometalation of sulfur-atom-substituted alkynes, such as alkynyl sulfones, sulfoxides, or sulfoximines as electron-deficient alkynes. Xie reported a copper-catalyzed carbomagnesiation of alkynyl sulfone to give the corresponding alkenylmagnesium intermediates (Scheme 2) [39–40]. Interestingly, the stereochemistry of the products was nicely controlled by the organomagnesium reagents and electrophiles employed. The reaction with arylmagnesium reagents provided alkenylmagnesium intermediate **1a**. The reaction of **1a** with allyl bromide provided *syn-*addition product **1b** in 70% yield while the reaction with benzaldehyde afforded *anti-*addition product **1c** in 59% yield. In contrast, allylmagnesiated intermediate **1d** reacted with benzaldehyde to give *syn*-addition product **1e** stereoselectively.

Marek reported copper-catalyzed carbometalation of alkynyl sulfoximines and sulfones using organozinc reagents of mild reactivity [41]. Various organozinc reagents can be used, irrespective of the identity of the organic groups, preparative protocols, and accompanying functional groups (Table 1). Similarly, Xie reported ethyl- or methylzincation of alkynyl sulfones [42] and Tanaka reported carbozincation of alkynyl sulfoxides [43–44].

**Table 1:** Copper-catalyzed carbozincation of alkynyl sulfoximines.

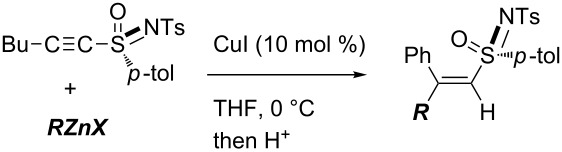		

Entry	***RZnX***	Yield

1	Et_2_Zn	82%
2	EtZnI^a^	90%
3	OctZnI^b^	55%
4	EtZnBr^c^	75%
5	iPrZnBr^c^	80%
6	PhZnBr^c^	85%
7	MeOCO(CH_2_)_3_ZnI^b^	55%
8	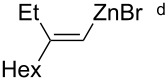	72%

^a^Prepared from Et_2_Zn and I_2_. ^b^Prepared from the corresponding alkyl iodide and zinc dust. ^c^Generated from the corresponding Grignard reagent and ZnBr_2_. ^d^Prepared from the corresponding vinyl iodide by iodine–lithium exchange and followed by transmetalation with ZnBr_2_.

**Scheme 3:**
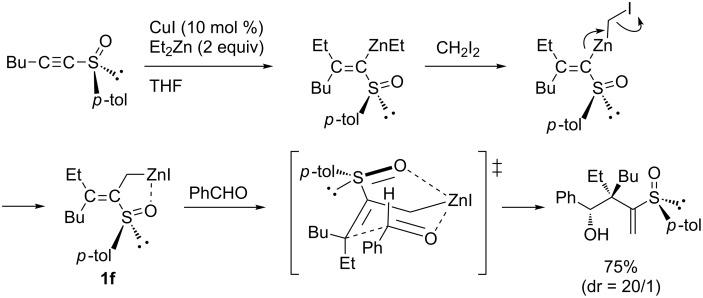
Copper-catalyzed four-component reaction of alkynyl sulfoxide with alkylzinc reagent, diiodomethane, and benzaldehyde.

Marek discovered efficient methods for the stereoselective synthesis of multisubstituted allylic zinc intermediates **1f** from alkynyl sulfoxides with organomagnesium or -zinc reagents (Scheme 3) [45–46]. It is noteworthy that they applied their chemistry to the preparation of various allylic metals [24–25,47–53] or enolates [54] from simple alkynes by carbocupration reactions.

**Scheme 4:**
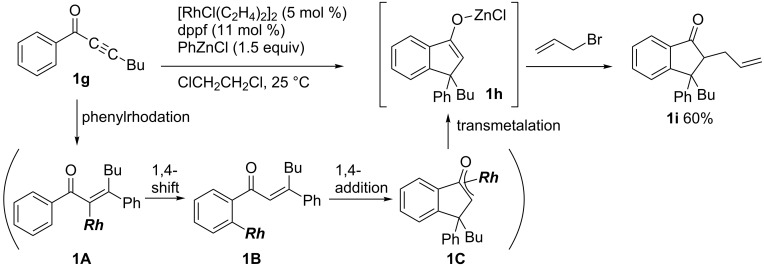
Rhodium-catalyzed reaction of aryl alkynyl ketones with arylzinc reagents.

Not only copper but also rhodium can catalyze carbometalation reactions. Hayashi applied carborhodation chemistry [55–58] to the reactions of aryl alkynyl ketones with arylzinc reagents, which provided enolates of indanones (Scheme 4) [59]. Phenylrhodation of **1g** first proceeds to form **1A**. A subsequent intramolecular 1,4-hydrogen shift gives **1B**, which smoothly undergoes an intramolecular 1,4-addition to yield **1C**. Finally, transmetalation from the phenylzinc reagent to rhodium enolate **1C** affords zinc enolate **1h**, which reacts with allyl bromide to give **1i** in 60% yield.

### Carbomagnesiation and carbozincation of alkynes bearing a directing group

**Scheme 5:**
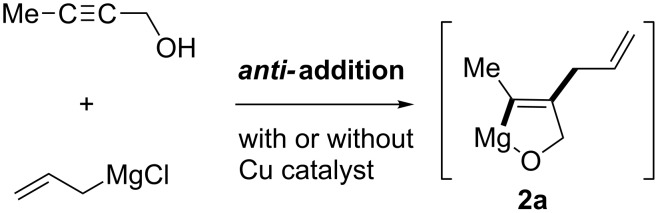
Allylmagnesiation of propargyl alcohol, which provides the *anti-*addition product.

**Scheme 6:**
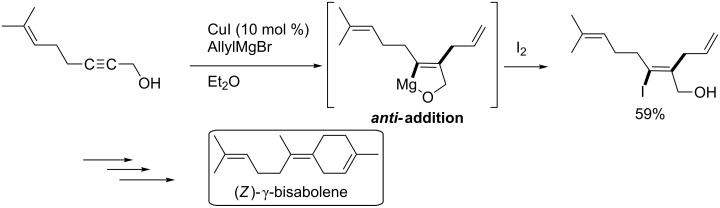
Negishi’s total synthesis of (*Z*)-γ-bisabolene by allylmagnesiation.

Directing groups have been utilized in successful carbometalation with high regio- and stereoselectivity. Classically, hydroxy groups on propargylic alcohols are used in uncatalyzed carbomagnesiation (Scheme 5) [60–61]. This addition proceeded in an *anti* fashion to give intermediate **2a**. The trend is the same in copper-catalyzed reactions of wide scope [62]. In 2001, Negishi applied copper-catalyzed allylmagnesiation to the total synthesis of (*Z*)-γ-bisabolene (Scheme 6) [63].

**Scheme 7:**
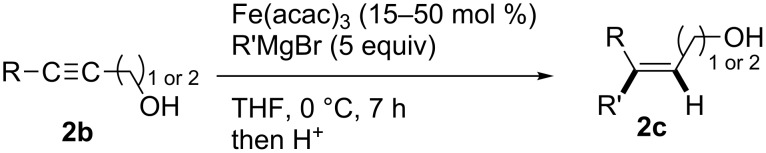
Iron-catalyzed *syn-*carbomagnesiation of propargylic or homopropargylic alcohol.

**Scheme 8:**
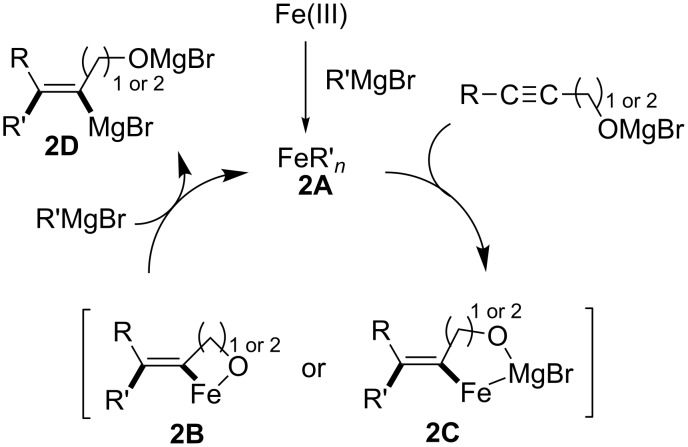
Mechanism of iron-catalyzed carbomagnesiation.

Recently, Ready reported an intriguing iron-catalyzed carbomagnesiation of propargylic and homopropargylic alcohols **2b** to yield *syn-*addition intermediates **2c** with opposite regioselectivity (Scheme 7) [64]. They assumed that the key organo-iron intermediate **2A** underwent oxygen-directed carbometalation to afford **2B** or **2C** (Scheme 8). Further transmetalation of vinyliron intermediate **2B** or **2C** with R′MgBr yielded the corresponding vinylmagnesium intermediate **2D**. Therefore, selective synthesis of both regioisomers of allylic alcohols can be accomplished by simply choosing transition-metal catalysts (Cu or Fe). Methyl-, ethyl-, and phenylmagnesium reagents could be employed for the reaction.

**Scheme 9:**
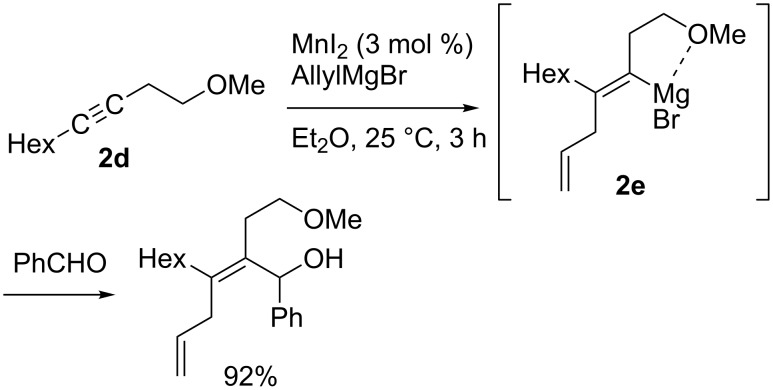
Regio- and stereoselective manganese-catalyzed allylmagnesiation.

**Scheme 10:**
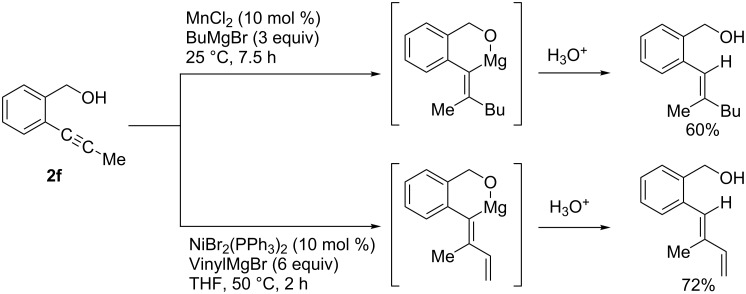
Vinylation and alkylation of arylacetylene-bearing hydroxy group.

Aside from the examples shown in Scheme 5 and Scheme 6, alkynes that possess a directing group usually undergo *syn-*addition. Oshima reported manganese-catalyzed regio- and stereoselective carbomagnesiation of homopropargyl ether **2d** leading to the formation of the corresponding *syn-*addition product **2e** (Scheme 9) [65]. The reaction of [2-(1-propynyl)phenyl]methanol (**2f**) also proceeded in a *syn* fashion (Scheme 10) [66–68].

**Scheme 11:**
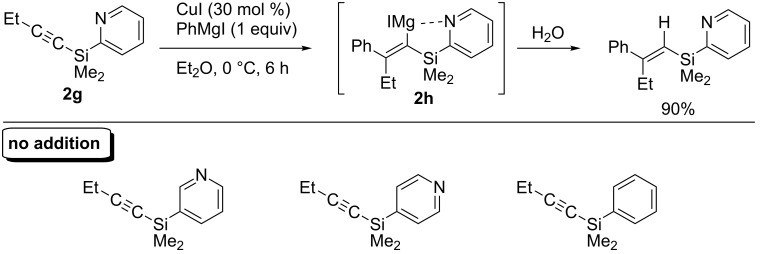
Arylmagnesiation of (2-pyridyl)silyl-substituted alkynes.

**Scheme 12:**
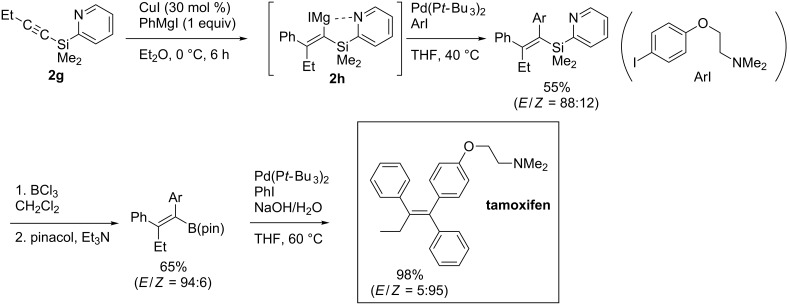
Synthesis of tamoxifen from **2g**.

In 2003, Itami and Yoshida revealed a concise synthesis of tetrasubstituted olefins from (2-pyridyl)silyl-substituted alkynes. The key intermediate **2h** was prepared by copper-catalyzed arylmagnesiation of **2g**, in which the 2-pyridyl group on silicon efficiently worked as a strong directing group (Scheme 11) [69]. Furthermore, they accomplished a short and efficient synthesis of tamoxifen from **2g** (Scheme 12). Notably, the synthetic procedure is significantly versatile and various tamoxifen derivatives were also prepared in just three steps from **2g**.

**Scheme 13:**
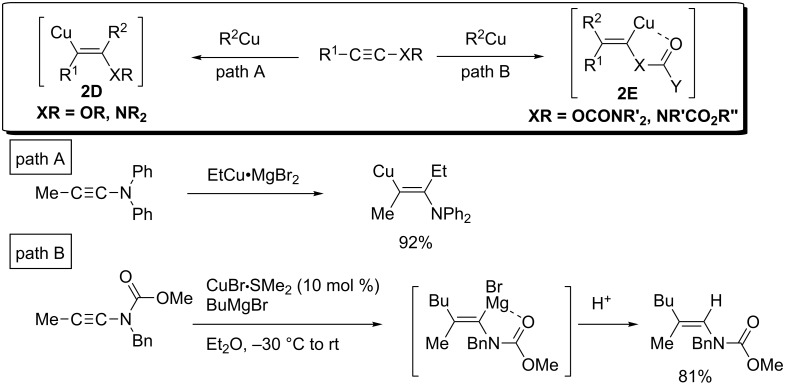
Controlling regioselectivity of carbocupration by attaching directing groups.

The directing effect dramatically changed the regioselectivity in the reactions of oxygen- or nitrogen-substituted alkynes. Carbocupration of these alkynes generally gives the vicinal product **2D** (copper locates at the β-position to the O or N) (Scheme 13, path A) [14,70–73]. On the other hand, the reversed regioselectivity was observed in the carbocupration of *O-*alkynyl carbamate and *N*-alkynyl carbamate, in which carbonyl groups worked as a directing group to control the regioselectivity to afford **2E** (Scheme 13, path B) [25,74–76].

**Scheme 14:**
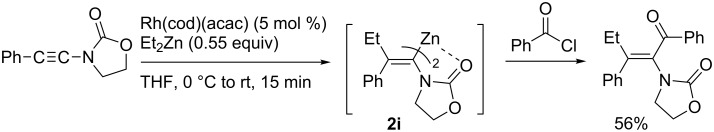
Rhodium-catalyzed carbozincation of ynamides.

In 2009, Lam reported the rhodium-catalyzed carbozincation of ynamides. The reaction smoothly proceeded under mild conditions to provide the corresponding intermediate **2i** regioselectively (Scheme 14) [77–78]. A wide variety of ynamides and organozinc reagents could be used for the reaction (Table 2).

**Table 2:** Scope of rhodium-catalyzed carbozincation of ynamide.

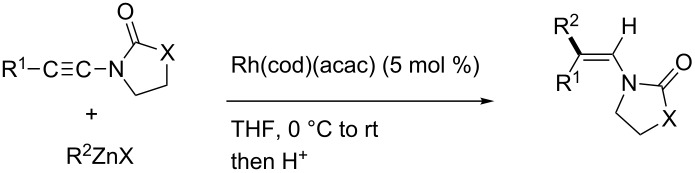					

Reagent	Product	Yield [%]	Reagent	Product	Yield [%]

Bn_2_Zn	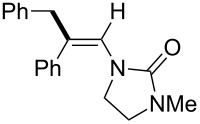	71	(*p*-FC_6_H_4_)_2_Zn	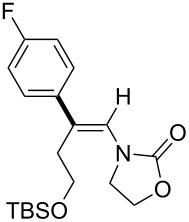	84
(vinyl)_2_Zn	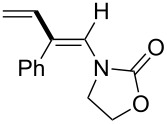	66	(phenylethynyl)_2_Zn	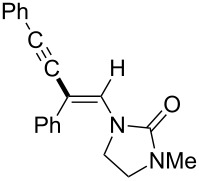	60
(2-propenyl)_2_Zn	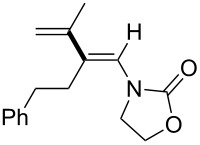	47	EtO_2_C(CH_2_)_3_ZnBr	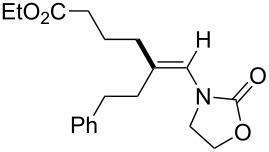	54
(2-thienyl)_2_Zn	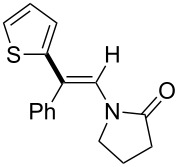	66	*m*-EtO_2_CC_6_H_4_ZnI	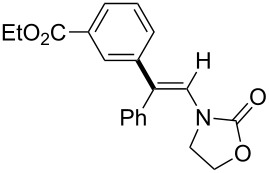	58

**Scheme 15:**
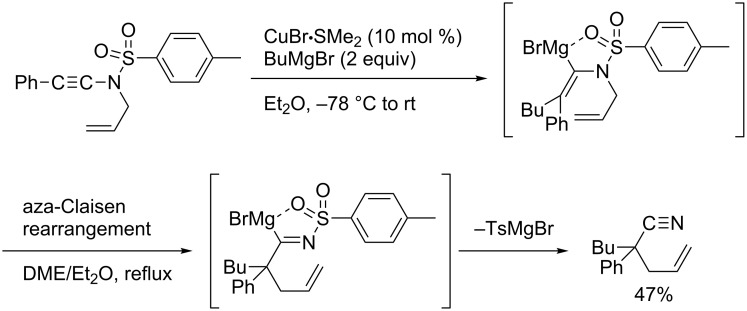
Synthesis of 4-pentenenitriles through carbometalation followed by aza-Claisen rearrangement.

Yorimitsu and Oshima reported an interesting transformation of ynamides to nitriles by a carbomagnesiation/aza-Claisen rearrangement sequence (Scheme 15) [79–80].

### Carbomagnesiation and carbozincation of cyclopropenes

**Scheme 16:**
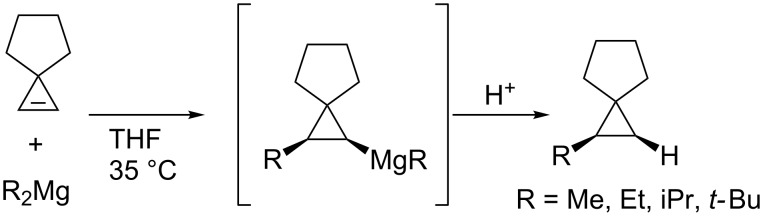
Uncatalyzed carbomagnesiation of cyclopropenes.

Increasing the reactivity of alkynes and alkenes is another strategy to achieve intermolecular carbometalation reactions. For example, strained alkenes are highly reactive toward carbometalation. The reactions of cyclopropenes took place without the aid of a metal catalyst (Scheme 16) [81].

**Scheme 17:**
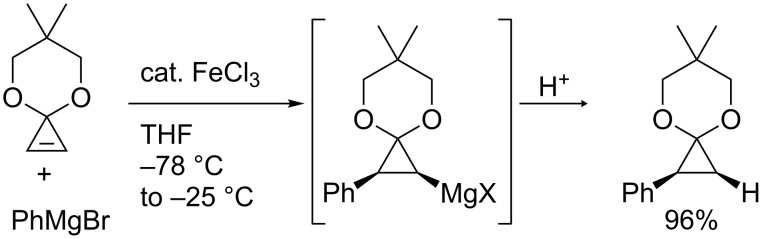
Iron-catalyzed carbometalation of cyclopropenes.

**Scheme 18:**
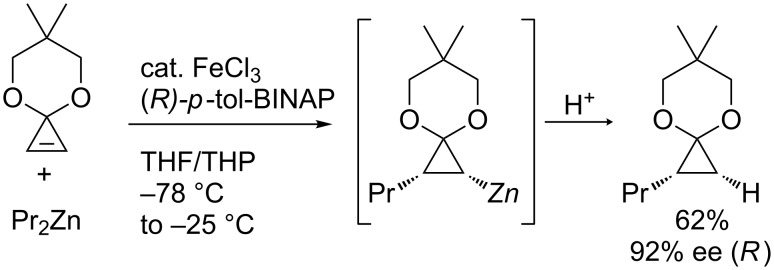
Enantioselective carbozincation of cyclopropenes.

Nakamura and co-workers discovered that an addition of iron salt enhanced the carbometalation of cyclopropenone acetal with organomagnesium and -zinc species (Scheme 17) and applied the reaction to enantioselective carbozincation (Scheme 18) [82–83]. The scope was wide enough to use phenyl-, vinyl-, and methylmagnesium reagents or diethylzinc and dipentylzinc reagents. It is noteworthy that the reaction in the absence of the iron catalyst did not proceed at low temperature and gave a complex mixture at higher temperature (up to 65 °C).

**Scheme 19:**
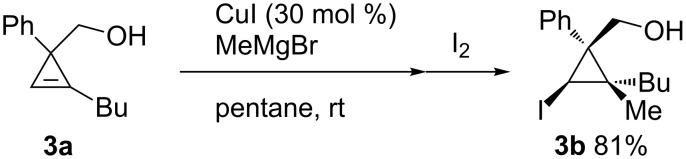
Copper-catalyzed facially selective carbomagnesiation.

**Scheme 20:**
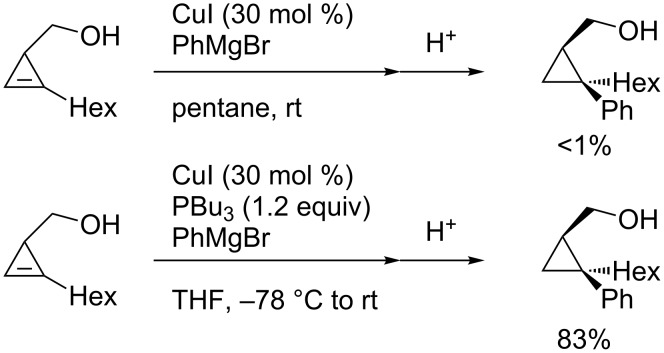
Arylmagnesiation of cyclopropenes.

A hydroxymethyl group also showed a significant directing effect in the copper-catalyzed reaction of cyclopropene **3a** with methylmagnesium reagent to afford **3b** with perfect stereoselectivity (Scheme 19) [84]. Not only methylmagnesium reagents but also vinyl- or alkynylmagnesium reagents could be employed. Although the arylation reaction did not proceed under the same conditions (Scheme 20, top), the addition of tributylphosphine and the use of THF as a solvent enabled the stereoselective arylmagnesiation with high efficiency (Scheme 20, bottom) [85]. Similarly, carbocupration reactions of 1-cyclopropenylmethanol and its derivatives using the directing effect of the hydroxymethyl group are also known [86–89].

**Scheme 21:**
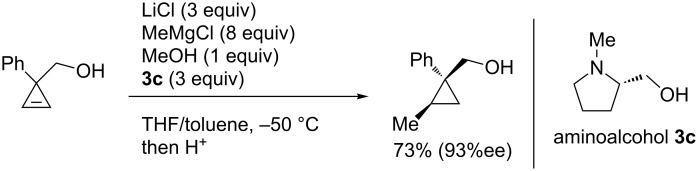
Enantioselective methylmagnesiation of cyclopropenes without catalyst.

**Scheme 22:**
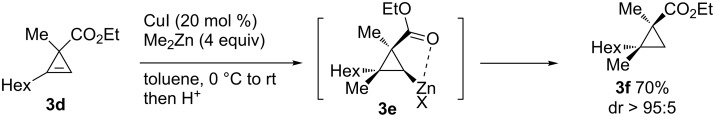
Copper-catalyzed carbozincation.

Notably, Fox reported the enantio- and stereoselective carbomagnesiation of cyclopropenes without the addition of transition metals (Scheme 21) [90]. The key to successful reactions is the addition of aminoalcohol **3c** and 1 equiv of methanol. In 2009, Fox et al. improved their copper-catalyzed carbometalation reactions of cyclopropenes by using functional-group-tolerable organozinc reagents (Scheme 22) such as dimethyl-, diethyl-, diphenyl-, diisopropyl-, and divinylzinc reagents [91]. Treatment of cyclopropene **3d** with dimethylzinc in the presence of a catalytic amount of copper iodide afforded organozinc intermediate **3e** and finally **3f** after protonolysis. In 2012, Fox reported the stereoselective copper-catalyzed arylzincation of cyclopropenes with a wider variety of arylzinc reagents [92]. The organozinc reagents were prepared by iodine/magnesium exchange and the subsequent transmetalation to zinc, and then used directly in one pot (Table 3).

**Table 3:** Sequential I/Mg/Zn exchange and arylzincation of cyclopropenes.

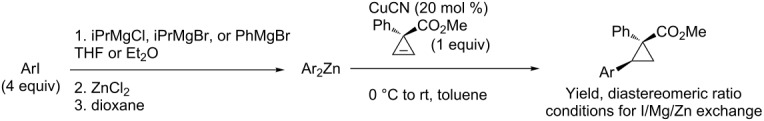					

Reaction step 1.	Product	Yield [%] (dr)	Reaction step 1.	Product	Yield [%] (dr)

iPrMgCl, Et_2_O, rt	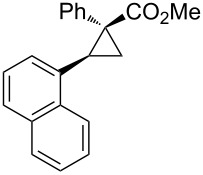	81 (>95:5)	PhMgBr, THF, −35 °C	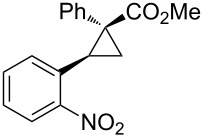	62 (95:5)
iPrMgCl, THF, −35 °C	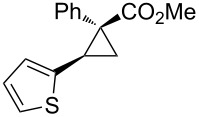	69 (>95:5)	PhMgBr, THF, −40 °C	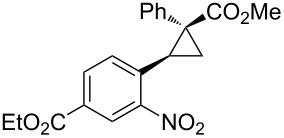	61 (>95:5)
iPrMgCl, Et_2_O, −35 °C	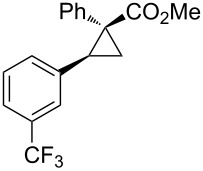	70 (>95:5)	iPrMgBr, THF, rt	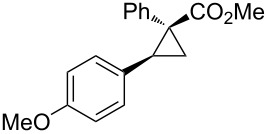	60 (88:12)
iPrMgCl, THF, −35 °C	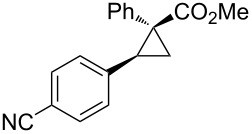	55 (91:9)			

**Scheme 23:**
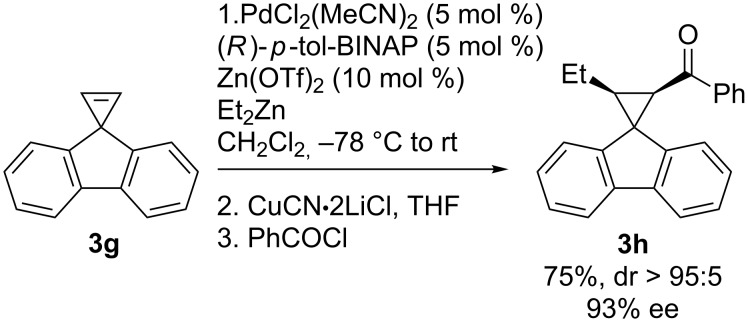
Enantioselective ethylzincation of cyclopropenes.

Lautens reported enantioselective carbozincation of alkenes using a palladium catalyst with a chiral ligand (Scheme 23) [93]. Treatment of **3g** with diethylzinc in the presence of catalytic amounts of palladium salt, (*R*)-tol-BINAP, and zinc triflate and subsequent quenching with benzoyl chloride afforded **3h** in 75% yield with 93% ee. The addition of zinc triflate may help the formation of a more reactive cationic palladium(II) species. Under similar conditions, Lautens also reported palladium-catalyzed carbozincation of oxabicycloalkenes [94].

**Scheme 24:**
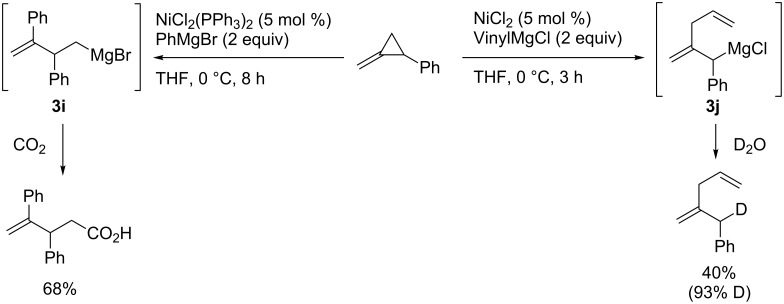
Nickel-catalyzed ring-opening aryl- and alkenylmagnesiation of a methylenecyclopropane.

**Scheme 25:**
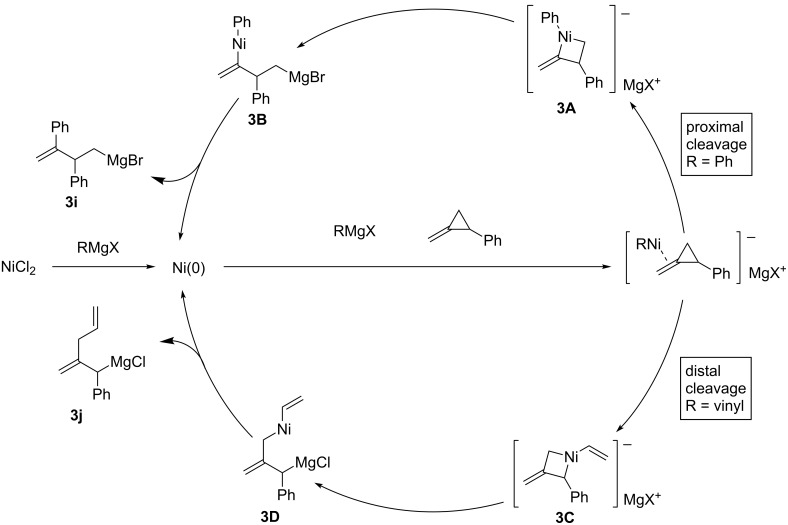
Reaction mechanism.

Terao and Kambe reported two types of intriguing ring-opening carbomagnesiations of a methylenecyclopropane that proceed through site-selective carbon–carbon bond cleavage (Scheme 24) [95]. The reaction pathways depended on the reagents used, i.e., the reaction with a phenylmagnesium reagent provided **3i** whereas the reaction with a vinylmagnesium reagent gave **3j**. The reaction mechanisms are shown in Scheme 25. They proposed that the carbon–carbon bond cleavage happened prior to the carbometalation reactions, which is different from other ring-opening reactions of cyclopropenes [96–97] where carbometalation is followed by carbon–carbon bond cleavage. Firstly, the oxidative addition of methylenecyclopropane to the reduced nickel(0) may yield **3A** or **3C**. The subsequent isomerization would proceed to form **3B** or **3D**, respectively, and then reductive elimination would afford the corresponding organomagnesium intermediate **3i** or **3j**.

### Carbomagnesiation and carbozincation of unfunctionalized alkynes and alkenes

**Scheme 26:**
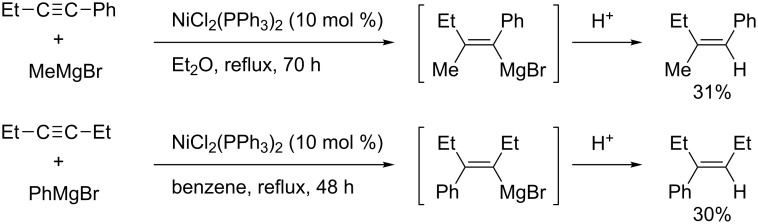
Nickel-catalyzed carbomagnesiation of arylacetylene and dialkylacetylene.

Carbomagnesiation and carbozincation of simple alkynes has been a longstanding challenge. In 1978, Duboudin reported nickel-catalyzed carbomagnesiation of unfunctionalized alkynes, such as phenylacetylenes and dialkylacetylenes (Scheme 26) [98]. Although this achievement is significant as an intermolecular carbomagnesiation of unreactive alkynes, the scope was fairly limited and yields were low.

**Scheme 27:**
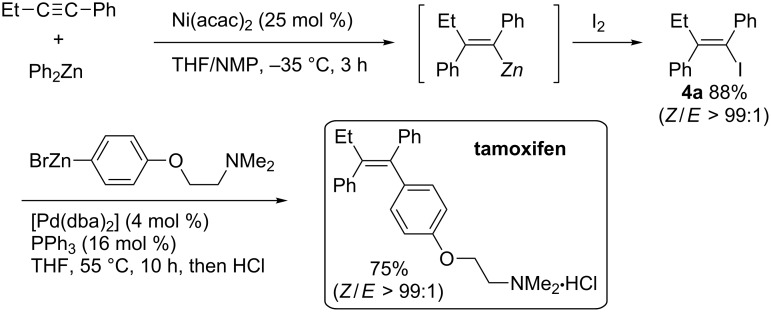
Nickel-catalyzed carbozincation of arylacetylenes and its application to the synthesis of tamoxifen.

**Scheme 28:**
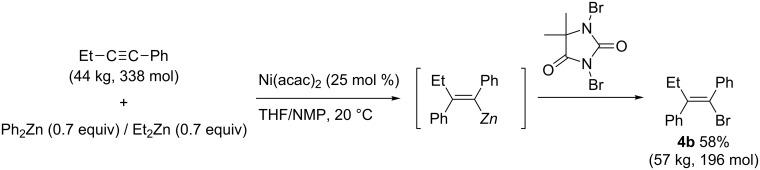
Bristol-Myers Squibb’s nickel-catalyzed phenylzincation.

In 1997, Knochel reported nickel-catalyzed carbozincation of arylacetylenes (Scheme 27) [99–100]. The reaction smoothly proceeded at –35 °C and exclusively produced tetrasubstituted (*Z*)-alkene **4a** in high yield. Not only diphenylzinc reagent but also dimethyl- and diethylzinc reagents were employed. Chemists at the Bristol-Myers Squibb company developed a scalable synthesis of (*Z*)-1-bromo-2-ethylstilbene (**4b**), a key intermediate of a selective estrogen-receptor modulator, using the modified Knochel’s carbozincation method (Scheme 28) [101]. It is noteworthy that the modified nickel-catalyzed reaction could be performed at 20 °C to afford 57 kg of the corresponding phenylated product (58% yield) from 44 kg of 1-phenyl-1-butyne.

Oshima reported manganese-catalyzed phenylmagnesiation of a wide range of arylacetylenes (Table 4) [102]. Notably, directing groups, such as *ortho*-methoxy or *ortho*-amino groups, facilitated the reaction (Table 4, entries 2 and 3 versus entry 4).

**Table 4:** Manganese-catalyzed arylmagnesiation of arylacetylenes.

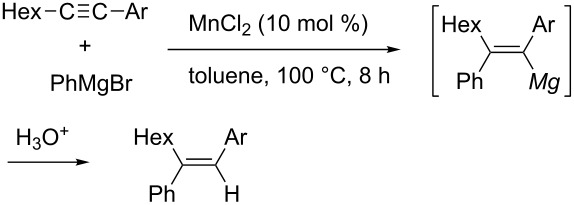		

Entry	Ar	Yield

1	Ph	66%
2	*o*-Me_2_NC_6_H_4_	94%
3	*o*-MeOC_6_H_4_	80%
4	*p*-MeOC_6_H_4_	38%
5	*o*-FC_6_H_4_	47%

**Scheme 29:**
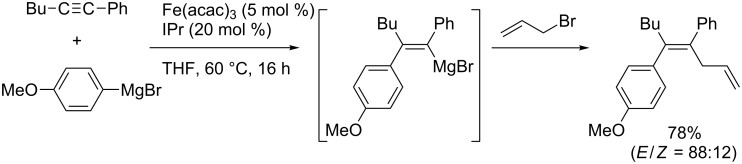
Iron/NHC-catalyzed arylmagnesiation of aryl(alkyl)acetylene.

Recently, iron and cobalt have been regarded as efficient catalysts for carbometalation of simple alkynes. Shirakawa and Hayashi reported that iron salts could catalyze arylmagnesiation of arylacetylenes in the presence of an *N*-heterocyclic carbene (NHC) ligand (Scheme 29) [103].

**Scheme 30:**
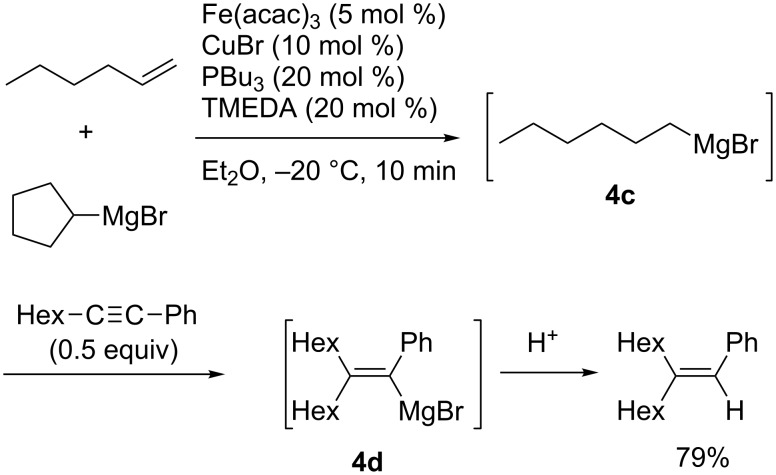
Iron/copper-cocatalyzed alkylmagnesiation of aryl(alkyl)acetylenes.

**Scheme 31:**
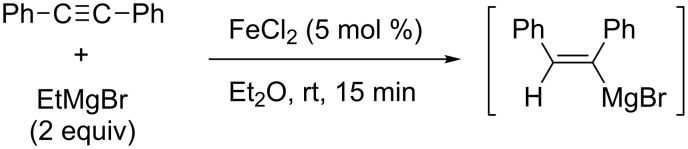
Iron-catalyzed hydrometalation.

In 2012, Shirakawa and Hayashi reported iron/copper cocatalyzed alkene–Grignard exchange reactions and their application to one-pot alkylmagnesiation of alkynes (Scheme 30) [104]. The exchange reactions proceeded through a β-hydrogen elimination–hydromagnesiation sequence to generate **4c**. The alkylmagnesiation reactions of 1-phenyl-1-octyne with **4c** provided the corresponding alkylated products **4d** exclusively without contamination by the hydromagnesiated products of alkynes. In contrast, Nakamura reported the iron-catalyzed hydromagnesiation of diarylacetylenes and diynes with ethylmagnesium bromide as a hydride donor without forming alkylated products (Scheme 31) [105].

**Scheme 32:**
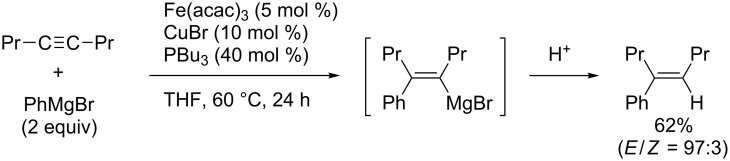
Iron/copper-cocatalyzed arylmagnesiation of dialkylacetylenes.

As shown in Scheme 32, carbomagnesiation of dialkylacetylene provided the corresponding arylated product only in low yield. Although Negishi reported ethylzincation [106], allylzincation [107], and methylalumination [108] with a stoichiometric amount of zirconium salt, the examples of transition-metal-catalyzed carbometalation of dialkylacetylenes were limited only to carboboration [109–110] and carbostannylation [111]. In 2005, Shirakawa and Hayashi reported iron/copper-cocatalyzed arylmagnesiation of dialkylacetylenes [112]. This is the first successful catalytic carbomagnesiation of dialkylacetylenes. Note that Ilies and Nakamura reported iron-catalyzed annulation reactions of various alkynes, including dialkylacetylenes with 2-biphenylylmagnesium reagents to form phenanthrene structures [113].

**Scheme 33:**
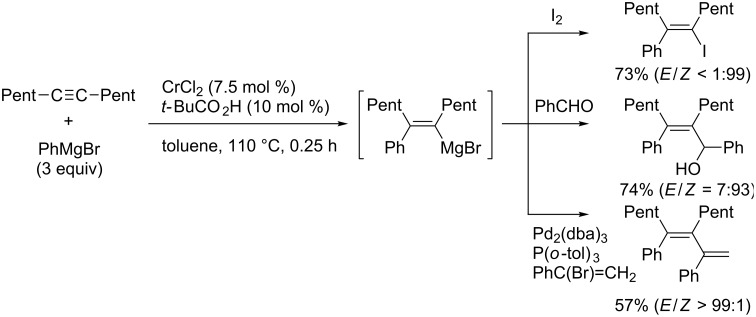
Chromium-catalyzed arylmagnesiation of alkynes.

In 2007, Yorimitsu and Oshima reported that chromium chloride could catalyze the arylmagnesiation of simple alkynes [114]. They found that the addition of a catalytic amount of pivalic acid dramatically accelerated the reaction (Table 5). Although the reason for the dramatic acceleration is not clear, the reaction provided various tetrasubstituted alkenes efficiently with good stereoselectivity (Scheme 33).

**Table 5:** Acceleration effect of additive on chromium-catalyzed arylmagnesiation.

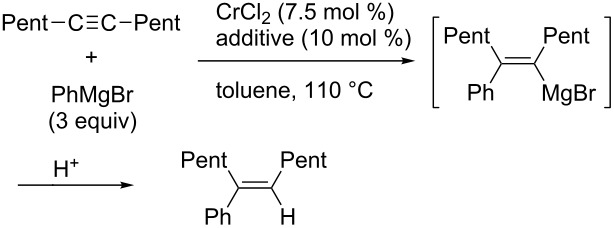			

Entry	Additive	Time	Yield (*E*/*Z*)

1	None	18 h	81% (91:9)
2	MeOH	2 h	77% (95:5)
3	MeCO_2_H	0.25 h	79% (>99:1)
4	PhCO_2_H	0.25 h	81% (>99:1)
**5**	***t-*BuCO_2_H**	**0.25 h**	**87% (>99:1)**

**Scheme 34:**
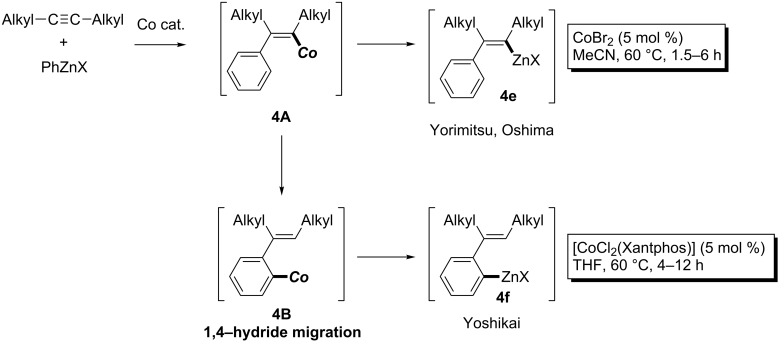
Cobalt-catalyzed arylzincation of alkynes.

A more versatile arylmetalation of dialkylacetylenes using arylzinc reagents in the presence of a cobalt catalyst was then reported by Yorimitsu and Oshima (Scheme 34, top) [115]. Treatment of dialkylacetylenes with arylzinc reagents in acetonitrile in the presence of a catalytic amount of cobalt bromide afforded the corresponding arylated intermediate **4e**. Further study by Yoshikai revealed that the use of Xantphos as a ligand totally changed the products [116]. Smooth 1,4-hydride migration from **4A** to **4B** happened to provide organozinc **4f** (Scheme 34, bottom). The versatile 1,4-migration reactions were widely applicable for the 1,2-difunctionalization of arenes.

**Scheme 35:**
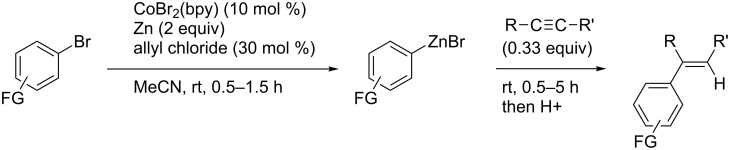
Cobalt-catalyzed formation of arylzinc reagents and subsequent arylzincation of alkynes.

In 2012, Gosmini reported similar cobalt-catalyzed arylzincation reactions of alkynes, which provided tri- or tetrasubstituted alkenes with high stereoselectivity [117]. Their catalytic system was dually efficient: the simple CoBr_2_(bpy) complex worked as a catalyst not only for arylzincation but also for the formation of arylzinc reagents (Scheme 35).

**Scheme 36:**
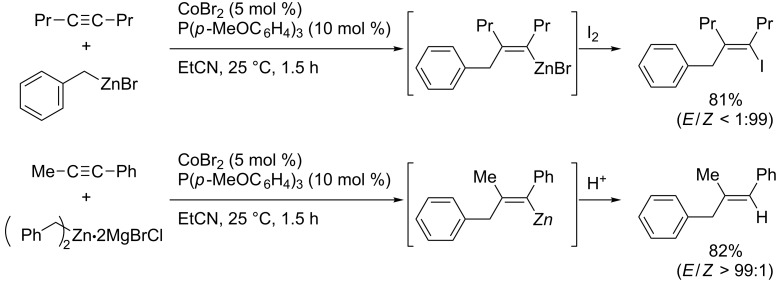
Cobalt-catalyzed benzylzincation of dialkylacetylene and aryl(alkyl)acetylenes.

**Scheme 37:**
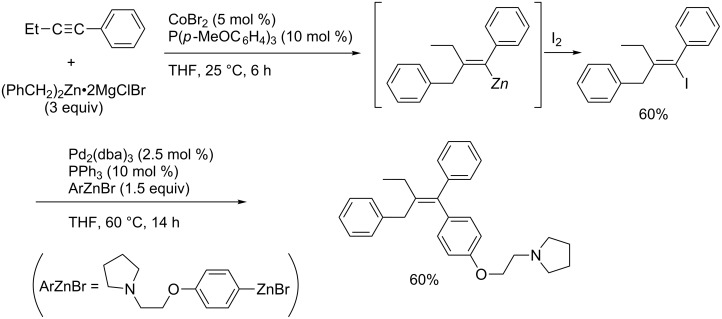
Synthesis of estrogen receptor antagonist.

**Scheme 38:**
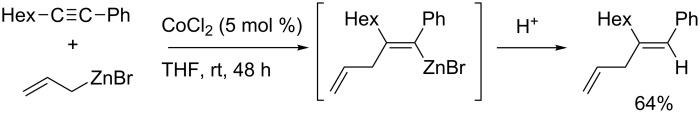
Cobalt-catalyzed allylzincation of aryl-substituted alkynes.

Yorimitsu and Oshima also accomplished benzylzincation of simple alkynes to provide allylbenzene derivatives in high yields (Scheme 36) [118]. For the reactions of simple dialkylacetylenes, benzylzinc bromide was effective (Scheme 36, top). On the other hand, dibenzylzinc reagent was effective for the reactions of aryl(alkyl)acetylenes (Scheme 36, bottom). They applied the reaction toward the synthesis of an estrogen-receptor antagonist (Scheme 37). Although the cobalt-catalyzed allylzincation reactions of dialkylacetylenes resulted in low yield, the reactions of arylacetylenes provided various tri- or tetrasubstituted styrene derivatives (Scheme 38) [119–120].

**Scheme 39:**
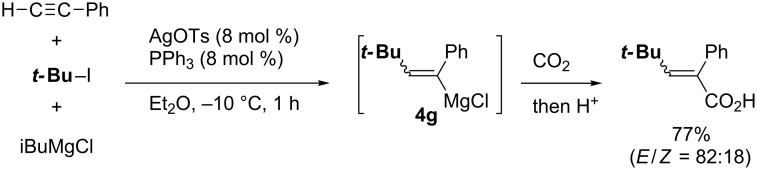
Silver-catalyzed alkylmagnesiation of terminal alkyne.

**Scheme 40:**
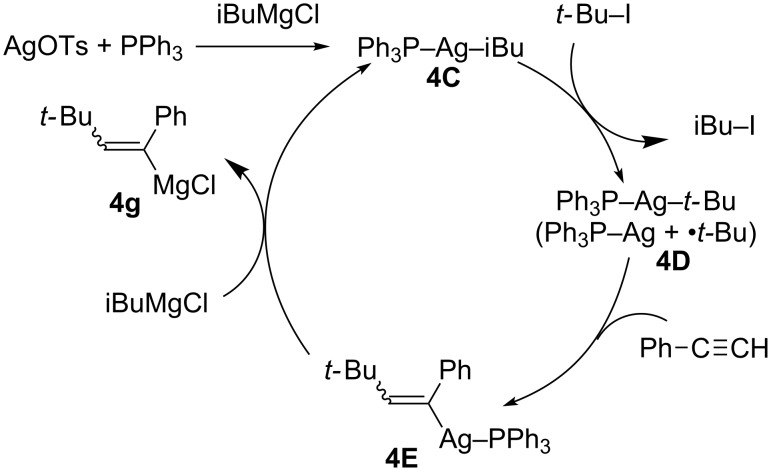
Proposed mechanism of silver-catalyzed alkylmagnesiation.

Kambe reported a rare example of silver catalysis for carbomagnesiation reactions of simple terminal alkynes (Scheme 39) [121–122]. They proposed that the catalytic cycle (Scheme 40) would be triggered by the transmetalation of AgOTs with iBuMgCl to afford isobutylsilver complex **4C**. Complex **4C** would react with *tert*-butyl iodide to generate *tert*-butylsilver intermediate **4D**. Carbometalation of terminal alkynes with **4D**, probably by addition of a *t-*Bu radical, would yield vinylsilver **4E**. Finally, transmetalation with iBuMgCl would give the corresponding vinylmagnesium intermediate **4g**. Due to the intermediacy of radical intermediates, the carbomagnesiation is not stereoselective.

**Scheme 41:**
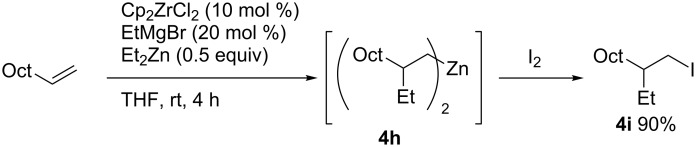
Zirconium-catalyzed ethylzincation of terminal alkenes.

In 2000, Negishi reported zirconium-catalyzed ethylzincation of 1-decene to provide dialkylzinc intermediate **4h** (Scheme 41) [123]. Intermediate **4h** reacted with iodine to provide alkyl iodide **4i** in 90% yield. The carbozincation reaction is cleaner and affords the corresponding products in high yields compared with the reported carbomagnesiation reactions [124–129].

**Scheme 42:**
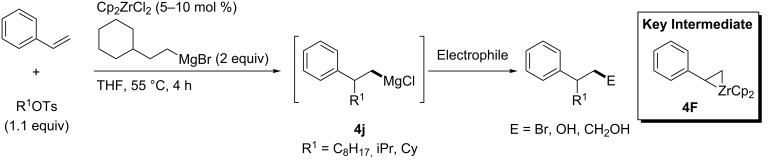
Zirconium-catalyzed alkylmagnesiation.

Hoveyda reported zirconium-catalyzed alkylmagnesiation reactions of styrene in 2001 by using primary or secondary alkyl tosylates as alkyl sources [130]. The reactions proceeded through zirconacyclopropane **4F** as a key intermediate to provide the corresponding alkylmagnesium compounds **4j**, which could be employed for further reactions with various electrophiles (Scheme 42).

**Scheme 43:**
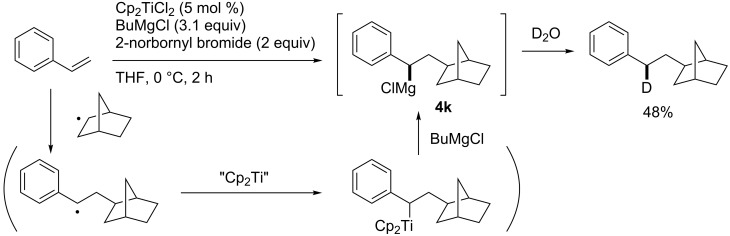
Titanium-catalyzed carbomagnesiation.

**Scheme 44:**
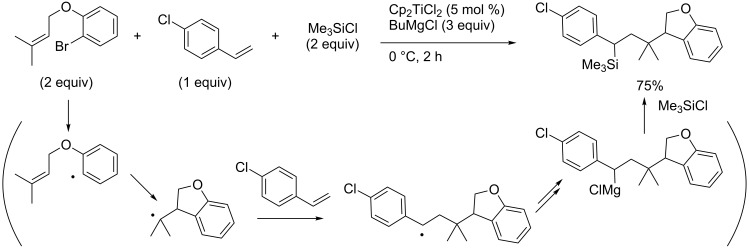
Three-component coupling reaction.

In 2004, Kambe reported titanocene-catalyzed carbomagnesiation, which proceeded through radical intermediates not metallacyclopropanes (Scheme 43) [131]. As a result, Hoveyda’s zirconium-catalyzed reactions provided homobenzylmagnesium intermediates **4j**, while Kambe’s titanium-catalyzed reactions afforded benzylmagnesium intermediates **4k**. Kambe applied the titanocene-catalyzed reaction to a three-component coupling reaction involving a radical cyclization reaction (Scheme 44).

**Scheme 45:**
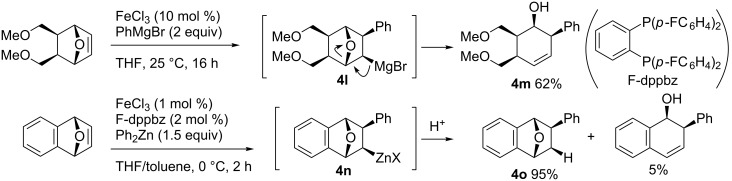
Iron-catalyzed arylzincation reaction of oxabicyclic alkenes.

Under Nakamura’s iron-catalyzed carbometalation reaction conditions (shown in Scheme 17), the reaction of oxabicyclic alkenes provided ring-opened product **4m** through a carbomagnesiation/elimination pathway (Scheme 45, reaction **4l** to **4m**) [82]. In contrast, the use of the 1,2-bis(diphenylphosphino)benzene (dppbz) ligand efficiently suppressed the elimination pathway to provide the corresponding carbozincation product **4o** in high yield (Scheme 45, reaction **4n** to **4o**) [132].

### Carbomagnesiation and carbozincation of allenes, dienes, enynes, and diynes

Interesting transformations were accomplished by the carbometalation of allenes, dienes, enynes, and diynes, since the resulting organometallic species inherently have additional saturation for further elaboration.

**Scheme 46:**
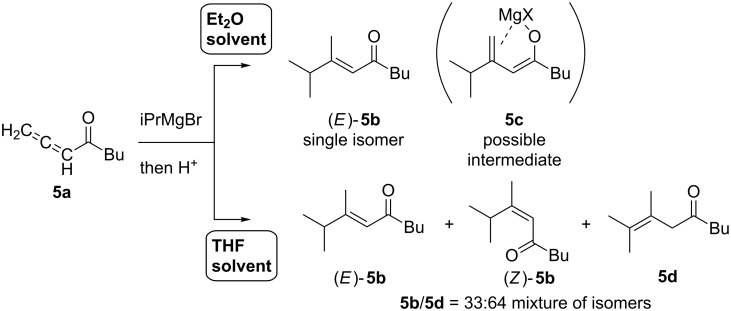
Reaction of allenyl ketones with organomagnesium reagent.

In 2002, Marek reported the reaction of allenyl ketones **5a** with organomagnesium reagents in the absence of a catalyst [133]. The reaction yielded α,β-unsaturated ketone (*E*)-**5b** as a single isomer in ether solution, while a mixture of isomers **5b** and **5d** was obtained in THF solution (Scheme 46). They proposed that the reason for the selectivity would be attributed to intermediate **5c**, which could stably exist in the less coordinative ether solution.

**Scheme 47:**
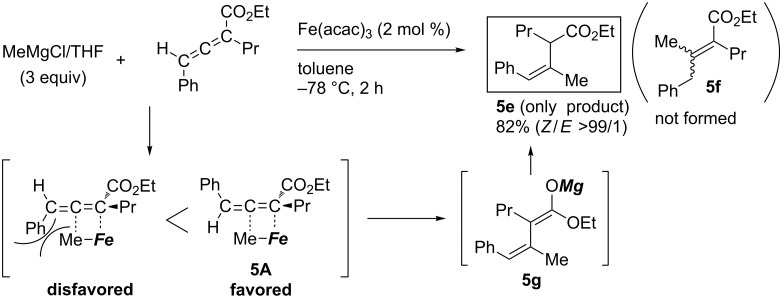
Regio- and stereoselective reaction of a 2,3-allenoate.

**Scheme 48:**
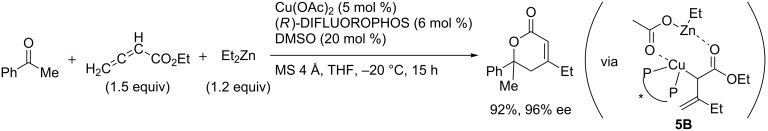
Three-component coupling reaction of 1,2-allenoate, organozinc reagent, and ketone.

Using an iron catalyst dramatically changed the trend of the addition product. Ma reported that treatment of a 2,3-allenoate with methylmagnesium reagent in the presence of a catalytic amount of iron catalyst exclusively gave the corresponding product **5e** (Scheme 47) [134]. Not only primary alkylmagnesium reagents but also secondary alkyl-, phenyl-, and vinylmagnesium reagents could be employed. Notably, α,β-unsaturated ester **5f** was not formed and the reaction was highly *Z*-selective. Ma explained that transition state **5A** would be favored because of the sterics to form intermediate **5g**. Independently, Kanai and Shibasaki reported copper-catalyzed enantioselective alkylative aldol reactions starting from 1,2-allenoate and dialkylzinc [135], which may proceed through carbometalation intermediates **5B** (Scheme 48).

**Scheme 49:**
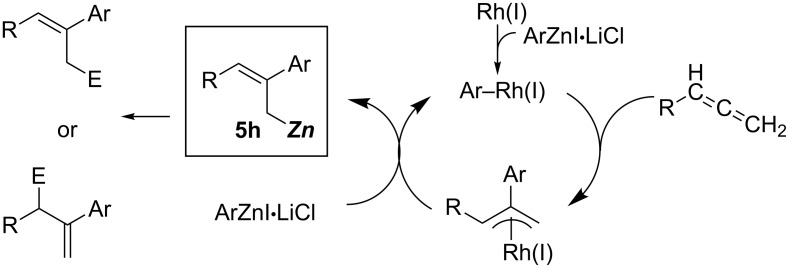
Proposed mechanism for a rhodium-catalyzed arylzincation of allenes.

**Scheme 50:**
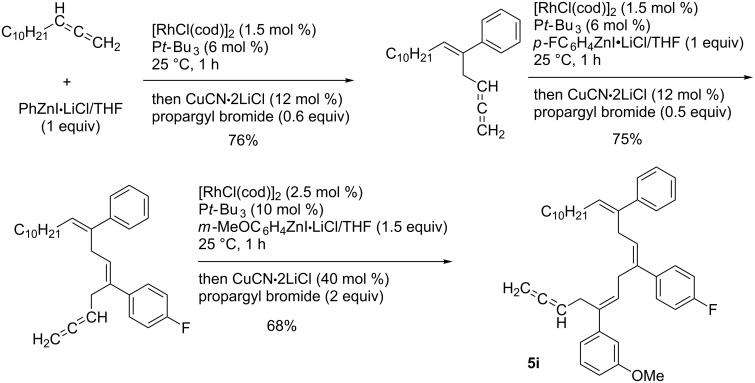
Synthesis of skipped polyenes by iterative arylzincation/allenylation reaction.

Yorimitsu and Oshima reported a rhodium-catalyzed arylzincation of simple terminal allenes that provided allylic zinc intermediates **5h** (Scheme 49) [136]. The resulting allylic zinc intermediates **5h** reacted with various electrophiles with high regio- and stereoselectivity. Thus, the reactions were applied to the synthesis of stereodefined skipped polyene **5i** via iterative arylzincation/allenylation reactions (Scheme 50).

**Scheme 51:**
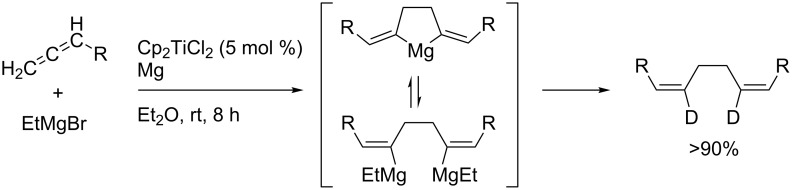
Synthesis of 1,4-diorganomagnesium compound from 1,2-dienes.

**Scheme 52:**
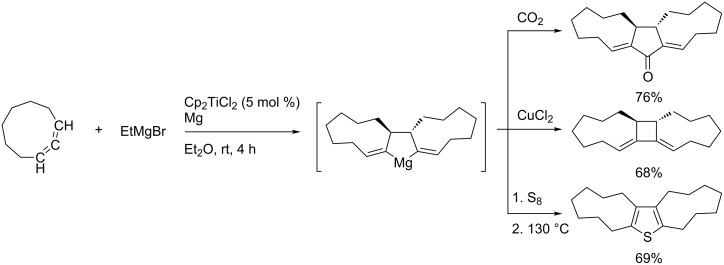
Synthesis of tricyclic compounds.

Zirconium-catalyzed dimerization of 1,2-dienes in preparation for the synthesis of useful 1,4-diorganomagnesium compounds from 1,2-dienes (Scheme 51) and its application to the synthesis of tricyclic compounds (Scheme 52) was reported [137–143].

**Scheme 53:**
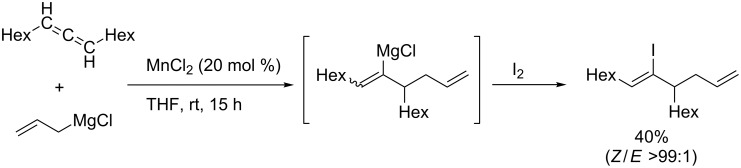
Manganese-catalyzed allylmagnesiation of allenes.

Manganese-catalyzed regioselective allylmetalation of allenes was reported (Scheme 53) [144]. The regioselectivity of the manganese-catalyzed addition reaction was opposite to that of the rhodium-catalyzed reactions, and vinylmagnesium intermediates were formed.

**Scheme 54:**
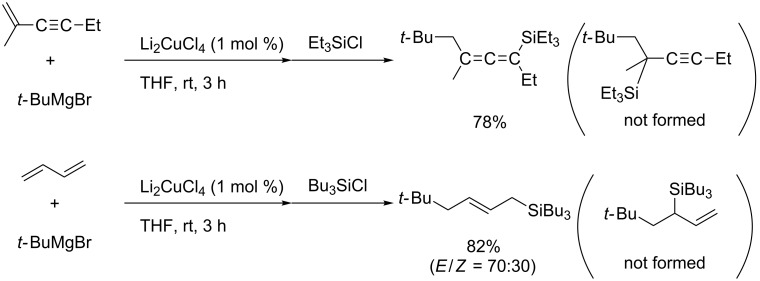
Copper-catalyzed alkylmagnesiation of 1,3-dienes and 1,3-enynes.

Although titanium-catalyzed allylmagnesiation of isoprene was reported in the 1970s, the scope of the reagents was limited to the allylic magnesium reagents [145–146]. Recently, Terao and Kambe reported copper-catalyzed regioselective carbomagnesiation of dienes and enynes using *sec*- or *tert*-alkylmagnesium reagents (Scheme 54) [147]. They assumed that the active species were organocuprates and that the radical character of carbocupration enabled bulky *sec*- or *tert*-alkylmagnesium reagents to be employed.

**Scheme 55:**
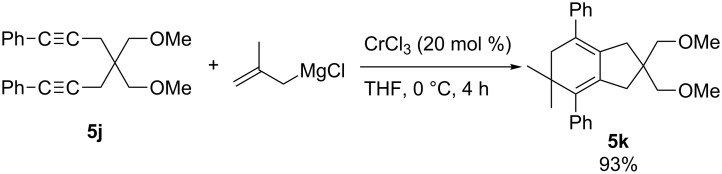
Chromium-catalyzed methallylmagnesiation of 1,6-diynes.

**Scheme 56:**
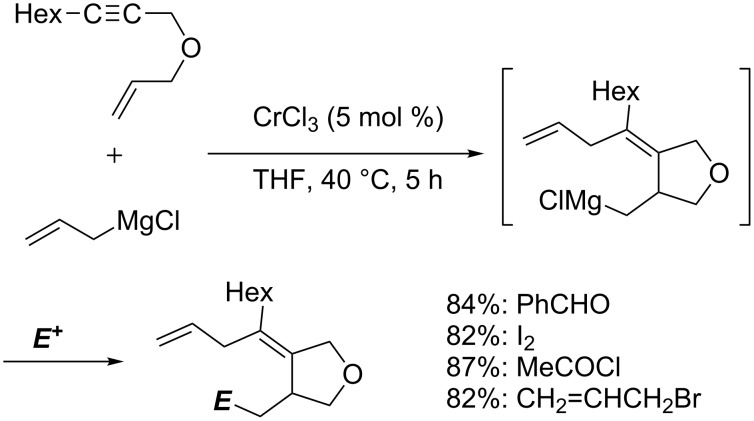
Chromium-catalyzed allylmagnesiation of 1,6-enynes.

**Scheme 57:**
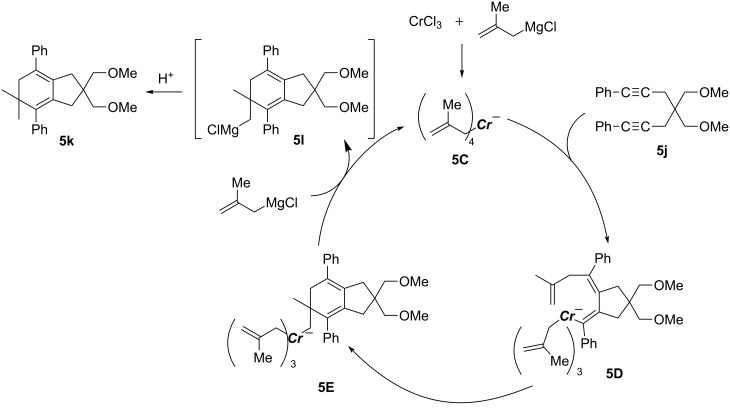
Proposed mechanism of the chromium-catalyzed methallylmagnesiation.

Chromium-catalyzed carbomagnesiation of 1,6-diyne (Scheme 55) [148] and 1,6-enyne (Scheme 56) [149] also provided interesting organomagnesium intermediates through cyclization reactions [150]. Treatment of 1,6-diyne **5j** with methallylmagnesium reagent in the presence of chromium(III) chloride afforded bicyclic product **5k** in excellent yield. In the proposed mechanism, the chromium salt was firstly converted to chromate **5C** by means of 4 equiv of methallylmagnesium reagent (Scheme 57). After the carbometalation followed by cyclization onto another alkyne moiety, vinylic organochromate **5D** would be then formed. Subsequent intramolecular carbochromation would provide **5E**, and finally transmetalation with methallylmagnesium reagent would give **5l** efficiently. The reaction of 1,6-enyne also proceeded through a tetraallylchromate complex as an active species (Scheme 56). However, the second cyclization did not take place.

## Conclusion

We have summarized the progress in transition-metal-catalyzed carbomagnesiation and carbozincation chemistry that has been made in the past 15 years. Despite the significant advances, there remains room for further improvements with regards to the scope of reagents, selectivity of the reaction, and information about the mechanisms, especially for alkenes as substrates. Further studies will surely provide powerful routes for functionalized multisubstituted alkenes and alkanes from simple alkynes and alkenes with high regio-, stereo-, and ultimately enantioselectivity.
